# Racial discrimination and allostatic load among First Nations Australians: a nationally representative cross-sectional study

**DOI:** 10.1186/s12889-020-09978-7

**Published:** 2020-12-07

**Authors:** Leah Cave, Matthew N. Cooper, Stephen R. Zubrick, Carrington C. J. Shepherd

**Affiliations:** 1 Telethon Kids Institute, The University of Western Australia, PO Box 855, West Perth, Western Australia 6872 Australia; 2 School of Population and Global Health, The University of Western Australia, 35 Stirling Highway, Perth, Western Australia 6009 Australia; 3 Centre for Child Health Research, The University of Western Australia, 35 Stirling Highway, Perth, Western Australia 6009 Australia; 4 Ngangk Yira Research Centre for Aboriginal Health & Social Equity, Murdoch University, 90 South Street, Murdoch, Western Australia 6150 Australia

**Keywords:** Racial discrimination, Allostatic load, Latent class analysis

## Abstract

**Background:**

Increased allostatic load is linked with racial discrimination exposure, providing a mechanism for the biological embedding of racism as a psychosocial stressor. We undertook an examination of how racial discrimination interacts with socioecological, environmental, and health conditions to affect multisystem dysregulation in a First Nations population.

**Methods:**

We conducted latent class analysis (LCA) using indicators of life stress, socioeconomic background, and physical and mental health from a nationally representative sample of Australian Aboriginal adults (*N* = 2056). We used LCA with distal outcomes to estimate the effect of the latent class variable on our derived allostatic load index and conducted a stratified analysis to test whether allostatic load varied based on exposure to racial discrimination across latent classes.

**Results:**

Our psychosocial, environmental, and health measures informed a four-class structure; ‘Low risk’, ‘Challenged but healthy’, ‘Mental health risk’ and ‘Multiple challenges’. Mean allostatic load was highest in ‘Multiple challenges’ compared to all other classes, both in those exposed (4.5; 95% CI: 3.9, 5.0) and not exposed (3.9; 95% CI: 3.7, 4.2) to racial discrimination. Allostatic load was significantly higher for those with exposure to racial discrimination in the ‘Multiple challenges’ class (*t* = 1.74, *p* = .04) and significantly lower in the ‘Mental health risk’ class (*t* = − 1.67, *p* = .05).

**Conclusions:**

Racial discrimination may not always modify physiological vulnerability to disease. Social and economic contexts must be considered when addressing the impact of racism, with a focus on individuals and sub-populations experiencing co-occurring life challenges.

**Supplementary Information:**

The online version contains supplementary material available at 10.1186/s12889-020-09978-7.

## Background

Racial discrimination is a well-established contributor to adverse physical health outcomes in ethnic/cultural minority populations [1–6]. Evidence detailing how racial discrimination affects physiology within the context of broader life circumstances is needed to encourage the development of informed strategies to prevent or eradicate racism in the settings where it occurs. This approach is particularly essential for Indigenous populations within high-income countries, where racial discrimination is often compounded by ongoing social marginalisation, land dispossession, and loss of sovereignty [7].

In Australian Aboriginal and Torres Strait Islander (hereafter referred to as ‘Aboriginal’) populations, where the pervasiveness of racial discrimination is well documented across legal, healthcare, educational and employment settings, [8–10] assessing the link between the psychosocial stressor of racism and physical health remains a challenge. To date, indicators of physical health most commonly comprise self- or carer-rated general health status, with few significant associations found [11–16]. Self-reported general health can be a misleading indicator of physical health, particularly for socially disadvantaged populations who may underreport or understate adverse health outcomes [17, 18]. Clinical biomarkers present a stronger indicator of underlying biological risk factors or early precursors of disease, and are typically unrecognised or not acknowledged by individuals responding to general health self-report measures. Individual biomarkers for allostatic load have been observed to be significantly higher in Aboriginal compared with non-Aboriginal populations [19] and studies have begun to investigate the association between racism and individual biomarkers in Aboriginal populations [20–24]. However, single biomarkers are specific to individual physiological systems and cannot capture the interactions and adaptions occurring between systems which lead to dysregulation.

Allostatic load is an indicator of multisystem physiological dysregulation resulting from over- or under-activation of systems designed to adapt to stress (i.e. metabolic, cardiovascular, immune, and neuroendocrine systems) [25]. Long periods of repeated exposure to psychosocial stressors such as racial discrimination are proposed to increase allostatic load, [26] promoting pathophysiology and ultimately increasing morbidity and mortality [27]. Increased allostatic load has also been linked to multiple other markers of stress and adversity, including socioeconomic status [27–31]. Associations between racial discrimination and allostatic load indices that comprise multiple biomarkers have been established in international studies [32–45]. Two of these studies were focused on Indigenous adults attending university in western Canada [39, 40]. In these Canadian studies, childhood experiences of racial discrimination and past year experiences of racial discrimination were both significantly associated with an allostatic load index comprised of seven biomarkers. The sample was dichotomised into groups with high and low cultural continuity, as defined by engagement in cultural practices and values related to their Indigenous cultural heritage at the time of data collection. Both studies observed that cultural continuity buffered the harmful effects of racial discrimination on allostatic load, with the association reduced to non-significance for those with high cultural continuity [39, 40]. Several studies have situated racism within the multiple intersecting stressors known to be implicated in heightened allostatic load but did so using mediation analysis or analysis of effect modification to investigate the role of specific variables measuring single environmental factors, such as emotional support, [37] social support, [43] racial background, [32, 33, 44] and socioeconomic status [32, 34]. Studies utilising this form of variable-centred analysis are less focused on examining the effects of multiple shared characteristics within population groups than on specified pathways between exposure and outcome variables. In contrast, person-centred analysis permits an examination of how racism is situated within—rather than isolated from—the complex reality of intersecting supports and stressors present within individual lives. Latent class analysis (LCA) is a form of person-centred analysis which identifies clusters or patterns of shared characteristics within groups of people and explores how outcomes of interest differ between these groups [46]. In this study, a latent class approach was used to determine whether exposure to racial discrimination could increase allostatic load for groups of people already experiencing many psychosocial stressors and adverse health, investigating whether racial discrimination can measurably increase allostatic load in the context of competing stressors and challenging circumstances.

This study undertakes the first examination of the role of racial discrimination on allostatic load in the context of broader socioecological, environmental, and health risk factors within a First Nations population. A more nuanced understanding of the role of racism is important in supporting intervention studies and policy approaches that seek to address the persistent impact of racism on population health disparities across the globe. We apply a latent class approach to nationally-representative data to first identify profiles of Aboriginal Australian adults defined by psychosocial stressors, environmental factors, health conditions, and mental health factors. We then examine the association between these profiles and an index of allostatic load. Finally, we examine the association between identified profiles and allostatic load after dichotomising the population into those with exposure to racial discrimination in the previous 12 months and those unexposed. Our primary objective was to determine the effect of exposure to racial discrimination on allostatic load in profiles of Aboriginal adults defined by stress-related psychosocial, environmental, and health factors.

## Methods

**Fig. 1:**
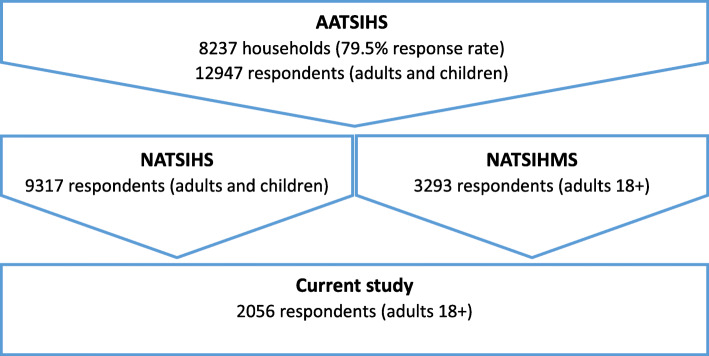
Selection of the study population

Data were from the National Aboriginal and Torres Strait Islander Health Survey (NATSIHS) and National Aboriginal and Torres Strait Islander Health Measures Survey (NATSIHMS) components of the Australian Bureau of Statistics’ (ABS) Australian Aboriginal and Torres Strait Islander Health Survey (AATSIHS) 2012–13. Full details of the data collection, recruitment, and sampling have been reported elsewhere [47, 48]. However, in brief, the AATSIHS selected a nationally representative sample of Aboriginal Australians via stratified multistage area sampling of private dwellings across remote, non-remote areas, and discrete Aboriginal and Torres Strait Islander communities nationally. The AATSIHS collected comprehensive health information from the Aboriginal population to obtain national benchmark information on health-related issues, collect biomarkers of chronic disease, and monitor trends in Aboriginal health. Selected adult members of the household consenting to participate undertook face-to-face interviews with trained interviewers. The AATSIHS core sample had a response rate of 79.5% fully or adequately responding households, culminating in 12,947 respondents aged 2 years and over. Adults aged 18 years and over (*n* = 8157) were invited to participate in the NATSIHMS, where biomedical data from blood and urine tests was collected. The current study analysed data from respondents within the NATSIHMS subset of the AATSIHS (*n* = 3293). The sample was weighted by the ABS to represent the national Aboriginal population as at 30 June 2011 (the estimated population benchmark from the 2011 Census of Population and Housing). Within the NATSIHMS subset, only individuals who also participated in the NATSIHS component of the AATSIHS were retained (*n* = 2060) as participant characteristics provided within this survey component informed explanatory variables within LCA. Four respondents were recorded as a ‘refusal’ in answering the racial discrimination measure and were excluded from further analysis, resulting in a final sample of 2056 respondents (Fig. 1).

The ABS has initiated several strategies to build and strengthen engagement with Aboriginal communities and organisations and to ensure best practice in the collection and dissemination of data from Aboriginal populations [49]. Led by the Centre of Excellence for Aboriginal and Torres Strait Islander Statistics, the ABS is guided by cultural protocols and procedures for engagement with Aboriginal peoples and communities developed in alignment with Indigenous data sovereignty principals [49, 50]. The current study received input and guidance on the study design and interpretation of findings from the Telethon Kids Institute Social Determinants of Aboriginal Health Community Advisory Group, an advisory group comprised of Aboriginal community members from the Perth region (Western Australia) established to ensure this research was directed and approved by the local Aboriginal community.

### Measures

#### Outcome variable

The outcome was an allostatic load index derived from 10 biomarkers selected based on their relevance in measuring physiological systems implicated in allostatic load and availability within the NATSIHMS [27]. Biomarkers frequently used in allostatic load studies represent physiological systems implicated in both the primary and secondary effects of stress exposure, including the neuroendocrine system (e.g. cortisol, epinephrine, dopamine); immune system (e.g. Interleukin-6, tumour necrosis factor-alpha, C-reactive protein); metabolic system (e.g. high density lipoprotein cholesterol, triglycerides, glucose, insulin, albumin, creatinine); cardiovascular/respiratory systems (e.g. systolic/diastolic blood pressure, peak expiratory flow, heart rate); and anthropomorphic measures (e.g. waist-to-hip ratio, body mass index) [27]. The physiological systems represented in this allostatic load index reflect secondary outcomes associated with longer-term stress exposure, as biomarkers constituting primary effects were not collected within the NATSIHMS (i.e. stress hormones, cortisol, epinephrine and norepinephrine or pro- and anti-inflammatory cytokines) [27, 51]. We have used a multi-systemic composite approach, including biomarkers representing several physiological systems to gain early indications of the secondary effects of stress [27, 51]. The biomarkers included in the current study were (cardiovascular system) systolic and diastolic blood pressure; (metabolic system) high density lipoprotein- (HDL) cholesterol, low density lipoprotein- (LDL) cholesterol, triglycerides, fasting glucose, plasma glycosylated haemoglobin (HbA1c), and albumin-creatinine ratio; (anthropomorphic) body mass index; and (immune system) C-reactive protein. To derive allostatic load, participants were given a value of ‘1’ for each biomarker that fell within the highest quartile of sample values (or the lowest quartile for HDL cholesterol), with these values then summed to a total score (index range 0–10; biomarkers weighted equally). This method has been applied to numerous studies to predict health outcomes, including later mortality, and to examine the variation produced by stress and related psychosocial factors [27, 52].

#### Racial discrimination

Respondents were asked whether they had felt they had been treated badly in the last twelve months because they were Aboriginal or Torres Strait Islander (yes/no).

#### Explanatory variables

Respondents within this dataset will have varying allostatic load scores based on their psychosocial, environmental, and physiological circumstances, and history. Factors that capture these circumstances have been included as explanatory variables in LCA as they will account for a portion of the variability in models of allostatic load. We selected a total of 15 explanatory variables for assessment in LCA — these factors were primarily related to stress, mental health, and physical health. Factors related to stress included low socioeconomic status (current unemployment, low education, area level disadvantage), financial difficulties, perceived social support, and death in the family. Factors related to mental health included psychological distress and a sense of control or mastery, while factors related to physical health included past or present health conditions, exercise, and having limited facilities for sanitation in the home. Geographic remoteness was also included as this can have both positive and negative implications for stress, mental health, and physical health due to either environmental and lifestyle stressors within metropolitan areas or challenges accessing services and infrastructure within remote areas. Substance use was initially included, although mean differences in allostatic load were minimal for substance use indicator variables so that stress and physical and mental health factors were ultimately prioritised for analysis.

Included variables spanned individual level (*n* = 10), family level (*n* = 3), and area level (*n* = 2) characteristics (Table 1). All explanatory variables were dichotomised with the reference category set to the low risk category, except area-level socioeconomic status which was a five-level categorical variable with the reference category set to the highest socioeconomic quintile.

**Table 1:** Explanatory variables and racial discrimination exposure

Individual level characteristics	n	%	95% CI	N^a^
Current or pre-existing kidney condition	138	6.8	5.2–8.4	2056
Current or pre-existing cardiac condition	836	40.2	37.2–43.2	2056
Current or pre-existing diabetes or related condition	693	31.7	28.9–34.4	2056
Current or pre-existing respiratory condition	857	45.6	42.5–48.6	2056
Psychological distress	617	30.5	27.7–33.4	2045
Low social support	129	12.7	9.9–15.4	947
Low sense of control	115	10.5	8.0–13.1	947
Did not meet exercise guidelines	507	53.2	49.0–57.5	949
Completed Year 11 or below	1087	51.1	47.9–54.2	1989
Unemployed or not in the labour force	1166	54.5	51.4–57.5	2056
Family level characteristics	n	%		N
Household financial stress	1122	54.8	51.7–58.0	1944
Family stressor - Death	798	50.0	46.5–53.5	1517
Limited facilities for sanitation at home	502	21.0	18.6–23.3	2054
Area level characteristics	n	%		N
1 – Most disadvantaged area	1233	54.5	51.4–57.6	2056
2	360	20.5	17.9–23.2	2056
3	205	10.9	9.0–12.8	2056
4	187	10.6	8.7–12.5	2056
5 – Least disadvantaged area	71	3.4	2.3–4.5	2056
Living in a remote area	1106	63.8	61.2–66.5	2056
Racial discrimination				
Exposure previous 12 months	292	14.6	12.4–16.8	2056

^a^Explanatory variables include missing data

#### Individual level characteristics

Individual level characteristics included health conditions, social and emotional wellbeing, exercise, education, and employment.

Health condition variables analysed (*n* = 4) included current or pre-existing kidney, cardiac, diabetes, and respiratory conditions. These condition categories were derived from 10 specific conditions reported by respondents. Respondents reporting kidney disease were classified with a ‘current or pre-existing kidney condition’, respondents reporting heart attack, heart failure, hypertensive disease or stroke were classified with a ‘current or pre-existing cardiac condition’, respondents reporting asthma, bronchitis, chronic sinusitis, or hayfever and allergic rhinitis were classified with a ‘current or pre-existing respiratory condition’, and respondents reporting diabetes mellitus (Type 1, 2 or unknown) were classified with a ‘current or pre-existing diabetes condition’. Each condition was considered present where the condition status was ‘ever told has condition, still current and long-term’; ‘ever told has condition, still current but not long-term’; ‘ever told has condition, not current’; or ‘not known or not ever told, but condition current and long-term’.

Social and emotional wellbeing variables analysed (*n* = 3) included the Kessler Psychological Distress Scale (Kessler-5), the Multidimensional Scale of Perceived Social Support (MSPSS) and an adaption of the Pearlin Mastery Scale. The Kessler-5 measure of psychological distress is derived from 5 items in the Kessler Psychological Distress Scale-10, designed to measure levels of negative emotional states in the four weeks prior to interview [53]. Responses were categorised according to the system used by the NATSIHS and dichotomised so that high/very high scores (12-25) indicate high levels of psychological distress while low scores (5-11) indicate low levels of psychological distress [54]. The MSPSS is a measure of perceived social support from family, friends, and significant others [55] and was derived from 6 items in the NATSIHS. A score of 6–18 was then generated so that a higher score indicated reduced social support. This scale was then dichotomised by categorising those in the top decile (at or above the 90th percentile) as having ‘low social support’. The Pearlin Mastery Scale measures the extent to which individuals feel in control over their life outcomes [56] and was derived from 7 items in the NATSIHS. A score of 7–21 was then generated so that a higher score indicated a reduced sense of control. This scale was then dichotomised by categorising those in the top decile as having a ‘low sense of control’.

Exercise was derived from a single item indicating whether the amount of exercise or physical activity respondents undertook in the previous week reached 150 min as per Australian National Physical Activity and Sedentary Behaviour guidelines for adults. Respondents who did not undertake 150 min or more were categorised as ‘did not meet exercise guidelines’. Education was derived from a single item indicating whether respondents had completed educational attainment up to Year 11 completion. Respondents who indicated they had completed up to Year 11 education or had no educational attainment were categorised as ‘Completed Year 11 or below’. Employment was derived from a single item indicating whether respondents were unemployed (looking for part-time or full-time work) or not in the labour force, using a categorisation of labour force status in which the Federal Government Community Development Employment Projects scheme was included in the category of ‘unemployed or not in the labour force’. Respondents who were unemployed or not in the labour force were categorised as ‘unemployed or not in the labour force’.

#### Family level characteristics

Family level characteristics included household financial stress, experiencing death as a family stressor, or having limited facilities for sanitation in the home.

Financial stress was derived from a single item indicating whether household members could raise $2000 in an emergency. Respondents who indicated they could not raise $2000 were categorised as experiencing ‘financial stress’. Death as a family stressor was derived from a single item indicating whether ‘death’ had occurred in the previous 12 months as a family stressor. Limited facilities for sanitation in the home was derived from a single variable indicating whether respondents’ homes had working facilities for washing people, washing clothes or bedding, preparing food, or sewerage facilities. Respondents who indicated that they had no working facilities for any one of these fundamental household tasks were categorised as having ‘limited facilities for sanitation at home’.

#### Area level characteristics

Area level characteristics included area level socioeconomic status and geographic remoteness. Area level socioeconomic status was determined from the Index of Relative Socioeconomic Disadvantage developed by the ABS [57]. This index ranks the relative level of disadvantage of areas using the attributes of all persons (Aboriginal and non-Aboriginal) in small areas, and includes measures of income, educational attainment, employment status, family composition, and employment categorisation (i.e. low skill occupations). For the purposes of this study, we have categorised the index values into quintiles. Geographic remoteness was determined according to the Australian Statistical Geographic Standard 2011 remoteness areas with non-remote areas encompassing ‘major cities of Australia’, ‘Inner regional Australia’ and ‘Outer regional Australia’, while remote areas encompassed ‘Remote Australia’ and ‘Very Remote Australia’. Respondents living in remote areas were categorised as ‘living in a remote area’.

#### Covariates

Age and gender were included as covariates in LCA models. Age was a continuous measure of respondent’s age in single years. Gender was categorised as female/male with male as the reference category.

### Data analysis

Multiple imputation by Chained Equations was conducted in SAS Enterprise Guide 7.15 to minimise potential bias due to missing biomarker data in the NATSIHMS (Additional file 1). The number of respondents with missing data ranged from 14 to 708 (0.7–34.4%) for each biomarker. Descriptive statistics and sample sizes for all biomarkers are included in Supplementary Table 1. Analysis was conducted on the final sample of 2056 respondents after imputation.

Preliminary analysis was run to generate the mean, standard error, and confidence intervals for our index of allostatic load. Percentage distributions of explanatory variables were calculated. Linear regression models, adjusted for age and gender, were used to test associations between each explanatory variable and allostatic load. These analyses were run in SAS Enterprise Guide Version 7.15.

LCA was used to identify health and psychosocial stress profiles of Aboriginal adults within our sample using the 15 explanatory variables. Statistical model fit was assessed using the Akaike Information Criterion (AIC), sample size adjusted Bayesian Information Criterion (BIC), log-likelihood, and entropy for models with 2–10 classes. After the model of best fit was identified, mean estimates of allostatic load were compared between classes using Bolck, Croon and Hagenaars adjusted proportional assignment to calculate distal outcome estimates [58, 59]. Finally, racial discrimination exposure was included as a grouping variable in LCA to dichotomise the sample so that mean estimates of allostatic load could be compared between respondents who reported racial discrimination exposure and respondents with no reported racial discrimination exposure within each latent class. Differences in mean allostatic load with exposure to racial discrimination are reported both within and between latent classes in Supplementary Table 2. All LCA was conducted using the LCA Stata Plugin and LCA_Distal_BCH Stata function [60, 61] in Stata Version 16.0.

All analysis incorporated the ABS-generated person-weights produced for the NATSINPAS dataset. Use of these weights allows us to use sample population data to estimate outcomes for the total in-scope population [47]. All data was accessed and analysis run within the ABS DataLab environment [62].

## Results

Summary statistics of the 2056 participants’ responses for the 15 explanatory variables are presented in Table 1; frequencies ranged from 6.8% (current or pre-existing kidney disease) to 63.7% (living in a remote area). The sample was comprised of 53.6% females, with ages ranging from 18 to 75 years (*M* = 41.5, *SD* = 21.2). Means, standard errors, and 75th percentile cut-offs for each of the ten biomarkers comprising the allostatic load risk index are presented in Table 2. The number of biomarkers per individual observed to fall into the high-risk category ranged from 1 to 10, with a mean of 2.4 (SD: 2.3; 95% CI: 2.4, 2.5).

**Table 2:** Allostatic load outcome variables

Biomarkers	Mean	SD	95% CI	*P*_75_	N
Systolic blood pressure	121.6	18.1	120.7–122.4	130.7	2056
Diastolic blood pressure	78.8	13.6	78.2–79.3	86.0	2056
Body mass index	29.6	9.1	29.3–29.9	33.9	2056
HDL cholesterol	1.2	0.3	1.2–1.2	0.9 ^b^	2056
LDL cholesterol	2.9	1.4	2.9–3.0	3.5	2056
Triglycerides	1.7	0.9	1.6–1.7	2.1	2056
Fasting plasma glucose ^a^	1.7	0.3	1.7–1.7	1.8	2056
HbA1c	5.8	1.4	5.8–5.9	5.9	2056
Albumin creatinine ratio ^a^	0.2	1.4	0.1–0.2	0.9	2056
C-reactive protein ^a^	1.1	1.4	1.1–1.2	2.0	2056
Allostatic load	Mean	SD	95% CI		N
Allostatic load risk index	2.4	2.3	2.3–2.5		2056

^a^Log transformed

^b^HDL cholesterol results within the lowest 25th percentile used to indicate risk (results from 0 to 0.9)

Increased age was significantly associated with increased allostatic load (*B* = 0.1, p = <.0001), while allostatic load did not differ between male and female participants (*B* = -0.02, *p* = .81). There were statistically significant differences in mean allostatic load across many of the explanatory variables included in LCA, after adjustment for age and gender (Table 3). The estimated prevalence of racial discrimination exposure within the previous 12 months was 14.6% (95% CI: 12.4,16.8). A statistically significant increase in mean allostatic load was observed in participants who reported racial discrimination, after adjustment for age and gender (*B* = 0.38, *p* = .02) (Table 3).

**Table 3:** Mean allostatic load by explanatory variables

Explanatory variables	Mean	SE	Β ^a^	95% CI	*P*-value
**Current or pre-existing kidney condition**					
Yes	3.3	0.2	0.39	0.03–0.74	0.03
No	2.4	0.1	Ref	…	…
**Current or pre-existing cardiac condition**					
Yes	3.1	0.1	0.53	0.48–0.59	<.0001
No	2.0	0.1	Ref	…	…
**Current or pre-existing diabetes or related condition**					
Yes	3.6	0.1	1.19	0.99–1.39	<.0001
No	1.9	0.1	Ref	…	…
**Current or pre-existing respiratory condition**					
Yes	2.5	0.1	−0.09	−0.27-0.09	0.31
No	2.4	0.1	Ref	…	…
**Psychological distress**					
Yes	2.4	0.1	−0.02	−0.21-0.17	0.84
No	2.5	0.1	Ref	…	…
**Low social support**					
Yes	2.4	0.2	0.09	−0.36-0.54	0.68
No	2.1	0.1	Ref	…	…
**Low sense of control**					
Yes	2.6	0.2	0.32	−0.07-0.71	0.10
No	2.1	0.1	Ref	…	…
**Did not meet exercise guidelines**					
Yes	2.3	0.1	0.16	−0.09-0.41	0.20
No	2.0	0.1	Ref	…	…
**Completed Year 11 or below**					
Yes	2.8	0.1	0.45	0.26–0.63	<.0001
No	2.1	0.1	Ref	…	…
**Unemployed or not in the labour force**					
Yes	2.7	0.1	0.43	0.24–0.61	<.0001
No	2.1	0.1	Ref	…	…
**Financial stress**					
Yes	2.7	0.1	0.77	0.60–0.94	<.0001
No	2.1	0.1	Ref	…	…
**Family stressor – Death**					
Yes	2.7	0.1	0.62	0.41–0.82	<.0001
No	2.0	0.1	Ref	…	…
**Limited facilities for sanitation at home**					
Yes	2.8	0.1	0.61	0.40–0.82	<.0001
No	2.3	0.1	Ref	…	…
**Index of Relative Socioeconomic Advantage and Disadvantage**					
1 – Most disadvantaged	2.6	0.1	1.27	0.79–1.75	<.0001
2	2.6	0.1	1.07	0.59–1.56	<.0001
3	2.3	0.2	0.87	0.35–1.39	0.001
4	2.2	0.2	0.78	0.25–1.30	0.004
5 – Least disadvantaged	1.3	0.2	Ref	…	…
**Living in a remote area**					
Yes	2.9	0.1	0.90	0.72–1.07	<.0001
No	2.2	0.1	Ref	…	…
**Racial discrimination exposure previous 12 months**					
Yes	2.7	0.1	0.28	0.04–0.51	0.02
No	2.4	0.1	Ref	…	…

^a^Beta estimates indicate group differences in mean allostatic load

### Latent class analysis

Fit indices for latent class models with 2 to 10 classes were generated (Table 4). The four-class model was chosen to represent the optimal number of classes based on an appropriate balance between the sample adjusted BIC, entropy, and log-likelihood estimates [46]. Item response probabilities for explanatory variables from the four-class model are presented alongside the probability of latent class membership both for respondents with and without exposure to racial discrimination in Table 5. The four-class model included (1) ‘Low risk’: characterised by low probabilities of current or pre-existing health conditions, mental health risk indicators, and low probabilities of socioeconomic risk factors; (2) ‘Challenged but healthy’: characterised by low probabilities of current or pre-existing health conditions, mental health risk indicators, but with moderate probability of experiencing death as a family stressor, and high probabilities of almost all socioeconomic risk factors including financial stress, living in a disadvantaged area, living in a remote area, unemployment, and low education; (3) ‘Mental health risk’: characterised by a high probability of psychological distress, moderate-high probability of financial stress and unemployment, high probability of a current or pre-existing respiratory condition and low probabilities of all other current or pre-existing health conditions; (4) ‘Multiple challenges’: characterised by high probabilities of current or pre-existing cardiac, respiratory, and diabetes or related conditions and a relatively high probability of a current or pre-existing kidney condition, moderate probabilities of psychological distress and experiencing death as a family stressor, and moderate-high probabilities of financial stress, low education, unemployment, and living in a disadvantaged area.

**Table 4:** Fit indices for one to ten latent class models

Number of classes	Log-likelihood	AIC	BIC (sample size adjusted)	Change in BIC	Entropy
1	−17,394	7715	7759		−2.06
2	−16,537	6039	6130	− 1629	0.73
3	−16,259	5522	5659	− 471	0.70
4	−16,141	5324	5508	− 151	0.70
5	−16,079	5237	5468	−40	0.70
6	−16,031	5179	5456	−12	0.68
7	−15,985	5126	5449	−7	0.68
8	−15,950	5093	5464	+ 15	0.70
9	−15,930	5091	5508	+ 44	0.71
10	−15,912	5092	5556	+ 48	0.71

**Table 5:** Latent class membership, item response probabilities, and mean allostatic load estimates by latent class and racial discrimination exposure

Latent class membership probabilities	Low risk	Challenged but healthy	Mental health risk	Multiple challenges
Racial discrimination exposure	0.220	0.188	0.231	0.362
No racial discrimination exposure	0.308	0.278	0.174	0.240
Item response probabilities				
Current or pre-existing diabetes or related condition	0.189	0.138	0.142	0.770
Current or pre-existing cardiac condition	0.258	0.179	0.311	0.861
Current or pre-existing respiratory condition	0.459	0.159	0.686	0.594
Current or pre-existing kidney condition	0.020	0.027	0.033	0.188
Psychological distress	0.096	0.248	0.655	0.357
Low social support	0.005	0.199	0.210	0.211
Low sense of control	0.000	0.000	0.237	0.147
Did not meet exercise guidelines	0.448	0.560	0.388	0.594
Family stressor – Death	0.459	0.674	0.519	0.630
Financial stress	0.157	0.875	0.678	0.586
Limited facilities for sanitation at home	0.020	0.464	0.152	0.208
Completed Year 11 or below	0.265	0.697	0.485	0.624
Unemployed or not in the labour force	0.109	0.711	0.733	0.740
Living in area of relative disadvantage	0.279	0.918	0.443	0.539
Living in a remote area	0.121	0.900	0.052	0.303
Mean allostatic load	Estimate (95% Confidence Intervals)			
Full sample (n = 2056)	1.67 (1.49, 1.85)	2.58 (2.41, 2.74)	1.75 (1.45, 2.04)	4.05 (3.84, 4.26)
Racial discrimination exposure (*n* = 292)	1.88 (1.35, 2.41)	2.85 (1.83, 2.74)	1.20 (0.61, 1.79)	4.48 (3.95, 5.00)
No racial discrimination exposure (*n* = 1764)	1.64 (1.45, 1.83)	2.67 (2.49, 2.85)	1.78 (1.46, 2.11)	3.95 (3.73, 4.18)

#### Latent classes and allostatic load

Mean allostatic load for each latent class was estimated after including the allostatic load risk index as a distal outcome in LCA in a model adjusted for age and gender (Table 5). Mean allostatic load was observed to vary significantly by latent class (*Z* = 301.7, p = <.001) (Table 6). The ‘Multiple challenges’ class was observed to have the highest mean allostatic load (4.05; 95% CI: 3.84, 4.26). This estimate was significantly higher than mean estimates for all other classes, while the estimate for the ‘Challenged but healthy’ class was significantly higher than the ‘Low risk’ (*B* = 0.44, SE = 0.07, p = <.0001) and ‘Mental health risk’ (*B* = 0.39, SE = 0.09, p = <.0001) classes. No significant difference in allostatic load was observed between the ‘Low risk’ and ‘Mental health risk’ classes.

**Table 6:** Wald chi-squared tests, allostatic load risk index

Full Sample		Estimate	SE	Wald statistic	P-value
Low risk	Challenged but healthy	−0.436	0.065	44.939	<.001
Low risk	Mental health risk	−0.048	0.109	0.192	ns
Low risk	Multiple challenges	−0.888	0.062	206.248	<.001
Challenged but healthy	Mental health risk	0.388	0.094	16.913	<.001
Challenged but healthy	Multiple challenges	− 0.453	0.0439	106.449	<.001
Mental health risk	Multiple challenges	− 0.841	0.093	82.170	<.001
	Omnibus test			301.737	<.001
Racial discrimination exposure					
Low risk	Challenged but healthy	−0.196	0.181	1.176	ns
Low risk	Mental health risk	0.447	0.306	2.136	ns
Low risk	Multiple challenges	−0.869	0.161	29.121	<.001
Challenged but healthy	Mental health risk	0.644	0.281	5.246	<.025
Challenged but healthy	Multiple challenges	−0.673	0.124	29.442	<.001
Mental health risk	Multiple challenges	−1.317	0.266	24.518	<.001
	Omnibus test			67.507	<.001
No racial discrimination exposure					
Low risk	Challenged but healthy	−0.488	0.069	50.119	<.001
Low risk	Mental health risk	−0.085	0.119	0.515	ns
Low risk	Multiple challenges	−0.881	0.067	173.425	<.001
Challenged but healthy	Mental health risk	0.402	0.103	15.315	<.001
Challenged but healthy	Multiple challenges	−0.393	0.0469	70.174	<.001
Mental health risk	Multiple challenges	−0.795	0.102	61.179	<.001
	Omnibus test			235.537	<.001

#### Latent classes, allostatic load and racial discrimination

Mean allostatic load for each latent class was again estimated in an extended latent class model that dichotomised respondents into those exposed and unexposed to racial discrimination in the previous 12 months before including the allostatic load index as a distal outcome (Table 5). This permitted mean allostatic load to be estimated in each latent class both for individuals with and without exposure to racial discrimination in the previous 12 months. Mean allostatic load varied significantly between latent classes both for those with (*Z* = 67.5, p = <.001) and those without exposure to racial discrimination (*Z* = 235.5, p = <.001) (Table 6).

Mean estimated allostatic load was significantly higher for those in the ‘Multiple challenges’ class with exposure to racial discrimination compared to those unexposed (*t* = 1.74, *p* = .04), while allostatic load was significantly lower for those in the ‘Mental health risk’ class with exposure to racial discrimination compared to those unexposed (*t* = − 1.67, *p* = .05) (Table 7). The ‘Multiple challenges’ class was observed to have the highest mean allostatic load compared with all other classes both for those with exposure to racial discrimination in the previous 12 months (4.48; 95% CI: 3.95, 5.00) and for those unexposed (3.95; 95% CI: 3.73, 4.18).

**Table 7:** Mean difference between those exposed and unexposed to racial discrimination, within latent classes

	Mean difference – exposed compared with unexposed	SE of difference between means	T statistic	P-value
Low risk	0.239	0.293	0.82	0.206
Challenged but healthy	−0.383	0.240	−1.47	0.072
Mental health risk	−0.585	0.350	−1.67	0.048
Multiple challenges	0.524	0.300	1.74	0.041

## Discussion

This study presents a first exploration of how patterns of psychosocial stressors, environmental and mental health factors, and health conditions within latent classes affect multisystem dysregulation in a First Nations population. We used LCA with distal outcomes to identify profiles of Aboriginal Australian adults defined by these factors, examine the association between these profiles and an index of allostatic load, and determine whether the association between latent classes and allostatic load differed for those exposed and unexposed to racial discrimination.

Our study observed that allostatic load was significantly increased for individuals with exposure to racial discrimination in the ‘Multiple challenges’ class compared to those unexposed to racial discrimination in this class. By contrast, allostatic load was significantly lower for those exposed to racial discrimination in the ‘Mental health risk’ class compared to those unexposed. No significant difference in allostatic load was observed between those exposed and unexposed to racial discrimination in other latent classes. The ‘Multiple challenges’ class was characterised by high proportions of individuals who reported experiencing death as a family stressor, financial stress, unemployment, and low education, alongside current or pre-existing diabetes and cardiac conditions. Overall, our results indicate that racial discrimination can measurably increase allostatic load for groups of Aboriginal adults with this profile of psychosocial stress and health vulnerability, suggesting that these individuals were still physiologically susceptible to the additional psychosocial stressor of racism.

This study confirms that while exposure to racial discrimination has an effect on the physiological dysregulation linked with allostatic load, this effect is significant only in select contexts. Allostatic load was significantly increased between those exposed and unexposed to racial discrimination within the ‘Multiple challenges’ class, significantly decreased between those exposed and unexposed to racial discrimination within the ‘Mental health risk’ class, and no significant differences were observed between those exposed and unexposed in the remaining classes. Multiple individual stressors were significantly associated with increased allostatic load after adjustment for age and gender, providing an indication of the variety of psychosocial stressors that can affect allostatic load. Overall, results from these analyses suggest that not all circumstances in which Aboriginal people live lend themselves to an increased vulnerability from the adverse physiological effects of racism. These findings underscore that we cannot easily summarise the broader circumstances of Aboriginal peoples’ lives or the continuing adaption of those lives to the adversities often present within them. Racial discrimination is frequently numbered among these adversities, yet when examining the impact of racism on physiological health it is worth broadening the scope of analysis to include, rather than adjust for, socioecological, environmental, and health factors. This is especially the case where these factors have the potential to moderate the body’s physiological response to stress.

Findings from this study show that Aboriginal adults living in high stress environments with low socioeconomic resources have a higher allostatic load than those living in a low stress environment with high socioeconomic resources. This is the case even in the absence of reported health conditions and psychological distress. Respondents in the ‘Challenged but healthy’ class were observed to have a significantly higher allostatic load than respondents in the ‘Low risk’ class in the total sample, both before and after grouping for racial discrimination exposure. Both class profiles indicated low proportions of those with pre-existing health conditions or psychological distress, although the ‘Challenged but healthy’ class had considerably higher proportions of respondents indicating financial stress, unemployment, low education, and area-level disadvantage compared to the ‘Low risk’ class. Our findings suggest that the financial stress and resource deprivation related to low socioeconomic status (SES) contributes to a higher allostatic load burden in Aboriginal adults with otherwise positive physical and mental health profiles. Strong and consistent associations between lower SES and allostatic load have been empirically established [29, 63] and various mediators of the association between SES and allostatic load have been identified [32]. Economic difficulties encountered during adulthood have also been observed to amplify allostatic load in those exposed to stressors during childhood and adolescence, suggesting that SES can have an additive effect where maladaptive physiologic patterns have already been established [64]. Mechanisms like this illustrate how allostatic load can be indicative of past stress, providing a marker of the cumulative physiological toll exacted on the body over time. These observations suggest that allostatic load provides both an indication of past challenges and a risk factor for future physiological response patterns [29]. Where individuals have faced many stressors or challenging environments in the past, these patterns are more likely to be physiologically damaging and disease-promoting [65]. Based on results from this study, it appears that SES-related determinants can contribute to increases in physiological dysregulation for Aboriginal adults and that measures of SES must be accounted for when examining allostatic load in this population.

Levels of allostatic load were significantly lower for respondents characterised by indicators of psychological distress (‘Mental health risk’) compared to respondents characterised by low SES (‘Challenged but healthy’). In fact, levels of allostatic load for those in the ‘Mental health risk’ class were comparable to those observed in the ‘Low risk’ class. Additionally, those in the ‘Mental health risk’ class with exposure to racial discrimination were observed to have significantly lower allostatic load compared to those who were unexposed. These findings suggest that the components of adverse mental health incorporated into LCA models did not completely explain an observable increase in allostatic load and that exposure to racial discrimination interacts with mental health to measurably reduce allostatic load. Strong evidence for a link between allostatic load and adverse mental health has been observed in international studies and allostatic load has been proposed as a likely predicter of adverse mental health in Aboriginal populations [19, 66, 67]. Currently, further research is required to fully determine the association between increased allostatic load and adverse mental health in Aboriginal Australian populations. Cortisol and cortisone levels measured from hair samples of Aboriginal young adults were not significantly associated with psychological distress in the Aboriginal Birth Cohort Study, [68] while no significant association was observed between an allostatic load index comprised of multiple biomarkers and depressive symptoms in recent research undertaken with Aboriginal adolescents and adults in Queensland, Australia [69]. However, the Queensland study observed that anhedonia and insomnia sub-scores were significantly associated with increased allostatic load in one of the study sites, suggesting that depression symptomology can influence allostatic load [69]. Our observation that psychological distress and low sense of control are not significantly associated with allostatic load in regression analysis adjusted for age and gender suggests a less marked association with these mental health symptoms. However, the implications of our observation that allostatic load is lower for those with a high mental health risk profile who report exposure to racial discrimination are less clear. Emerging evidence from Aboriginal populations indicates that chronic exposure to psychosocial stressors can lead to suppression of the hypothalamic-pituitary-adrenal (HPA) axis measured via lower cortisol, cortisone, and a blunted cortisol awakening response [24, 68, 70]. Although a broader set of biomarkers were incorporated into our allostatic load index, the combination of stressors present within our mental health risk profile in addition to racial discrimination exposure may be indicative of this type of physiological response.

The strengths of this study include the novel investigation of allostatic load, racial discrimination and psychosocial, environmental and health factors in a large nationally representative sample of Australian Aboriginal adults. As allostatic load is conceptualised as an indicator of the cumulative effects of stress and adversity, our study design ensured that a broad range of factors were accounted for when examining the link between racial discrimination and this measure of physiological dysregulation. Alongside these strengths, our study encountered some limitations. First, our study was cross-sectional and could not account for interactions between racial discrimination, psychosocial, environmental and health factors, and allostatic load over time. We are unable to determine whether exposure to racial discrimination had occurred prior to the previous 12 months, or the frequency and duration of this exposure. Neither are we able to determine the temporal sequence of many explanatory variables included in LCA, racial discrimination exposure and the development of physiological dysregulation captured by the index of allostatic load. Second, a critical component within the literature on life and environmental stress concerns perceptions of stress [71]. Respondents in this study were unable to indicate the presence or degree of stress they perceived in response to individual measures included as explanatory factors in LCA. Third, the measure of racial discrimination utilised in this study was a single-item measure that has not been methodologically validated. Racism is a complex phenomenon and multi-item measures have been shown to be more reliable and able to capture this complexity [72]. Validated, multi-item racial discrimination measures have been developed for Aboriginal populations (e.g. the Measure of Indigenous Racism Experiences) [73] and would more accurately reflect the experience of racism for these populations. Fourth, although the biomedical data collected in this study over 2012–13 represents the most recent and comprehensive available in a nationally representative Aboriginal population dataset, these data are now several years old. Despite this, there have been few significant changes in the social, economic, and health circumstances of Aboriginal children and families across Australia since the AATSIHS data were collected. Accordingly, we strongly believe the data continue to be a relevant and useful resource for examination of the circumstances of Aboriginal and Torres Strait Islander peoples. Finally, many individual biomarkers included in the allostatic load index used within this study are predictive of the current or previous health conditions reported by respondents that were used in latent class formation. For those in the ‘Multiple challenges’ class, levels of allostatic load are likely primarily driven by the high proportion of respondents reporting the presence of these conditions.

## Conclusions

We observed that allostatic load was significantly increased for those exposed to the psychosocial stressor of racism in a profile of Aboriginal adults already experiencing multiple challenges and adverse health. Our findings also indirectly imply that Aboriginal individuals with fewer challenges, greater resources, or differing life circumstances (e.g. living in a remote area) were more physiologically resilient to the additional stressor of racial discrimination. This finding does nothing to diminish the importance of responding to racial discrimination as a social determinant of health, rather it highlights that the specific conditions of Aboriginal people’s lives must be accounted for when attempting to understand the link between racism and physiological health. The harmful effects of racism on Aboriginal health are modifiable and a stronger understanding of the circumstances in which they occur can encourage the development of evidence-based prevention strategies to mitigate their onset and continuation. Prevention strategies may take the form of intervention programs with linked evaluations or policy approaches which cut across multiple systems (e.g. healthcare, education, housing) to address the impact of racism on national health disparities.

## Supplementary Information

**Additional file 1.** Detailed description of multiple imputation.

**Additional file 2.** STROBE statement. Checklist of reporting guidelines for observational studies (cross-sectional).

**Additional file 3: Table S1.** Complete cases and imputed values for individual biomarkers and allostatic load risk index.

**Additional file 4: Table S2.** Mean difference in allostatic load between those exposed and unexposed to racial discrimination, within and between class comparisons.

## Data Availability

The data that support the findings of this study are available from the Australian Bureau of Statistics but restrictions apply to the availability of these data, which were used under license for the current study, and so are not publicly available. Data are however available from the authors upon reasonable request and with permission of the Australian Bureau of Statistics.

## Abbreviations

AIC: Akaike Information Criterion
ACR: Albumin-creatinine ratio
AATSIHS: Australian Aboriginal and Torres Strait Islander Health Survey
ABS: Australian Bureau of Statistics
BIC: Bayesian Information Criterion
CI: Confidence interval
HDL: High density lipoprotein
Kessler-5: Kessler Psychological Distress Scale
LCA: Latent class analysis
LDL: Low density lipoprotein
MSPSS: Multidimensional Scale of Perceived Social Support
NATSIHMS: National Aboriginal and Torres Strait Islander Health Measures Survey
NATSIHS: National Aboriginal and Torres Strait Islander Health Survey
HbA1c: Plasma glycosylated haemoglobin
SE: Standard error
SD: Standard deviation
