# Supplementary material for: Racial discrimination and allostatic load among First Nations Australians: a nationally representative cross-sectional study
Source: BMC Public Health. 2020 Dec 7;20:1881. doi: 10.1186/s12889-020-09978-7 (PMC7720631; doi:10.1186/s12889-020-09978-7)
Supplement: Supplementary file 1 — Additional file 1. Detailed description of multiple imputation. [file 12889_2020_9978_MOESM1_ESM.docx]

**Additional file 1**

Multiple imputation by Chained Equations (MICE) was used to minimise potential bias due to missing biomarker data in the NATSIHMS. The number of respondents with missing data ranged from 14-708 for each biomarker (Supplementary Table 1). Five imputed datasets were generated with all biomarkers listed for imputation and ABS person-weights included as the weighting variable.

Pooled distal outcome estimates and standard errors were calculated from estimates generated by the five imputed datasets both for outcomes from results overall and from results grouped by racial discrimination exposure. Wald test estimates and standard errors comparing mean differences in allostatic load between latent classes were pooled from output generated by the five imputed data sets. The Z-test was then used to determine whether mean differences were statistically significant between latent classes. This process was repeated for both results overall and results grouped by exposure to racial discrimination. Finally, mean differences in allostatic load between those with and without exposure to racial discrimination within each latent class were compared using a one-tailed t-test based on pooled distal outcome estimates and standard deviations calculated from pooled standard errors and sample sizes within each group.
