# Supplementary material for: Racial discrimination and allostatic load among First Nations Australians: a nationally representative cross-sectional study
Source: BMC Public Health. 2020 Dec 7;20:1881. doi: 10.1186/s12889-020-09978-7 (PMC7720631; doi:10.1186/s12889-020-09978-7)
Supplement: Supplementary file 3 — Additional file 3: Table S1. Complete cases and imputed values for individual biomarkers and allostatic load risk index. [file 12889_2020_9978_MOESM3_ESM.docx]

Supplementary Table 1: Allostatic load outcome variables

|  | Complete cases | | | Imputed | | |
| --- | --- | --- | --- | --- | --- | --- |
| Biomarkers | Mean | SD | N | Mean | SD | N |
| Systolic blood pressure | 122.1 | 19.4 | 1949 | 121.6 | 18.1 | 2056 |
| Diastolic blood pressure | 79.3 | 11.7 | 1949 | 78.8 | 13.6 | 2056 |
| Body mass index | 29.3 | 7.1 | 1922 | 29.6 | 9.1 | 2056 |
| HDL cholesterol | 1.2 | 0.3 | 2042 | 1.2 | 0.3 | 2056 |
| LDL cholesterol | 2.9 | 0.9 | 1348 | 2.9 | 1.4 | 2056 |
| Triglycerides | 1.7 | 1.0 | 1386 | 1.7 | 0.9 | 2056 |
| Fasting plasma glucose^1^ | 1.7 | 0.3 | 1386 | 1.7 | 0.3 | 2056 |
| HbA1c | 6.0 | 1.5 | 2036 | 5.8 | 1.4 | 2056 |
| Albumin creatinine ratio^1^ | 0.4 | 1.6 | 1908 | 0.2 | 1.4 | 2056 |
| C-reactive protein^1^ | 1.3 | 1.1 | 2042 | 1.1 | 1.4 | 2056 |
| Allostatic load |  |  |  |  |  |  |
| Allostatic load risk index | 3.23 | 2.15 | 1152 | 2.4 | 2.3 | 2056 |

^1^ Log transformed
