# Supplementary material for: Racial discrimination and allostatic load among First Nations Australians: a nationally representative cross-sectional study
Source: BMC Public Health. 2020 Dec 7;20:1881. doi: 10.1186/s12889-020-09978-7 (PMC7720631; doi:10.1186/s12889-020-09978-7)
Supplement: Supplementary file 4 — Additional file 4: Table S2. Mean difference in allostatic load between those exposed and unexposed to racial discrimination, within and between class comparisons. [file 12889_2020_9978_MOESM4_ESM.docx]

Supplementary Table 2: Mean difference in allostatic load between racism exposure and no exposure, within and between latent class comparisons

| No racial discrimination exposure | Racial discrimination exposure | | Mean difference | SE of difference between means | T statistic | P-value |
| --- | --- | --- | --- | --- | --- | --- |
| Class 1 | | **Class 1** | 0.239 | 0.293 | 0.82 | 0.206 |
| Class 2 | | **Class 2** | -0.383 | 0.240 | -1.47 | 0.072 |
| Class 3 | | **Class 3** | -0.585 | 0.350 | -1.67 | 0.048 |
| Class 4 | | **Class 4** | 0.524 | 0.300 | 1.74 | 0.041 |
| Class 1 | | **Class 2** | -0.791 | 0.291 | -2.718 | 0.003 |
| Class 1 | | **Class 3** | 0.092 | 0.325 | 0.283 | 0.389 |
| Class 1 | | **Class 4** | -2.075 | 0.300 | -6.917 | <.001 |
| Class 2 | | **Class 3** | 0.500 | 0.298 | 1.676 | 0.047 |
| Class 2 | | **Class 4** | -1.667 | 0.270 | -6.170 | <.001 |
| Class 3 | | **Class 4** | -2.752 | 0.326 | -8.440 | <.001 |
